# High-stretch, tendon-driven, fiber-reinforced membrane soft actuators with multiple active degrees of freedom

**DOI:** 10.1038/s44172-023-00139-3

**Published:** 2024-02-23

**Authors:** Nick Sholl, Kamran Mohseni

**Affiliations:** 1 Department of Mechanical and Aerospace Engineering, University of Florida, Gainesville, FL 32611 USA; 2 Department of Electrical and Computer Engineering, University of Florida, Gainesville, FL 32611 USA

**Keywords:** Mechanical engineering, Actuators, Soft materials

## Abstract

Most soft actuators with multiple active degrees of freedom do not take advantage of the full extensibility of elastomer. Here we introduce a technique for better utilizing this extensibility for more versatile soft actuators. Embedded tendons that slide through channels within an inflatable, fiber-reinforced elastomer membrane enable active control of the membrane’s geometry at high elastomer stretches, bringing its functionality close to that of a natural hydrostatic skeleton. We demonstrate this using an initially planar, tendon-driven, fiber-reinforced membrane actuator with a single fluid cavity that can actively extend, contract, bend in multiple directions, and grasp when inflated. Most notably, the same membrane stretches to nearly three times its initial length directly along the path of a sliding tendon while performing these motions. Two such membranes are used on a robotic platform to walk with the gait of a velvet worm using a fixed mass of air, turn, climb a ramp, and navigate uneven terrain.

## Abstract (web summary)

Sholl and Mohseni design and synthesise soft actuators with multiple active degrees of freedom by introducing sliding polymer filaments (tendons) driven by an external servomotor within inflatable, fiber-reinforced elastomer membranes. These structures mimic the broad motility of a natural hydrostatic skeleton, exploiting the full extensibility of the elastomer matrix.

## Introduction

Soft roboticists are often inspired by the utility and versatility of hydrostatic skeletons^1^, particularly a subset called muscular hydrostats^2^. These constant-volume arrays of interwoven muscle fibers and connective tissue provide both actuation and adjustable structural support for appendages such as cephalopod arms, elephant trunks, and the human tongue. They are capable of extension, contraction, bending, and torsion with actively adjustable stiffness in each of these actuation modes, allowing for an exceptionally large and diverse range of three-dimensional motion without the need for any rigid structural support^3^. Octopuses, in particular, have learned to use the muscular hydrostats in their arms for both continuum (e.g., bend propagation^4^) and quasi-articulated (e.g., fetching^5^) actuation, highlighting the remarkable functionality that would be possible in a soft robot that properly replicates a muscular hydrostat. This has spurred a push within the soft robotics community to create cephalopod-inspired appendages (e.g., refs. ^6–9^) that can move in a similar fashion.

**Fig. 1:**
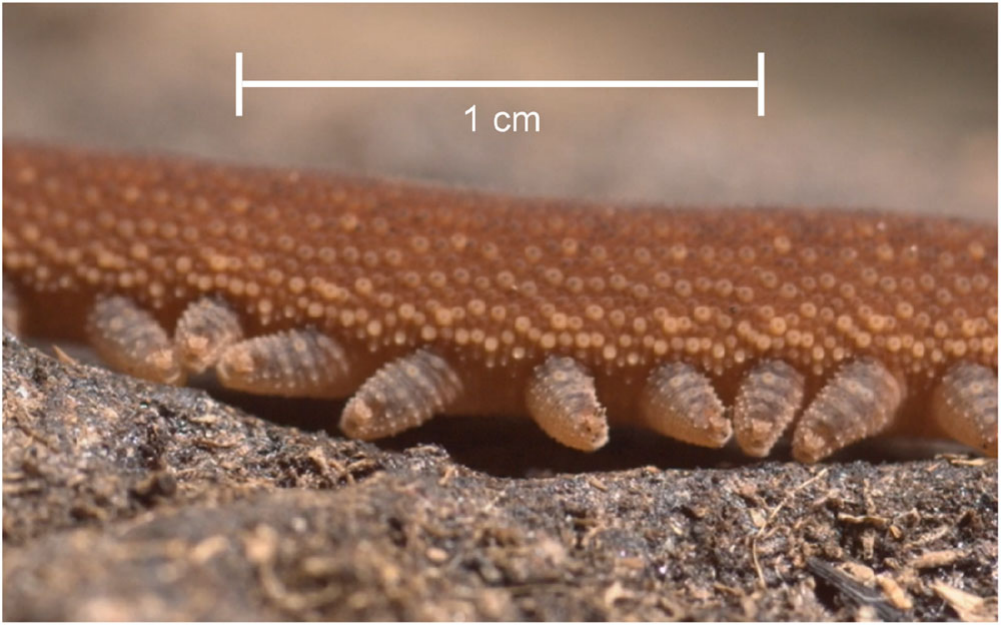
Velvet worm (*Principapillatus hitoyensis*) legs while walking. The scale bar shown is approximate and based on measurements of other animals of the same species. Photo reproduced with permission from Dr. Ivo de Sena Oliveira and Dr. Alexander Baer.

The broader category of hydrostatic skeletons is capable of many of the same motions as a muscular hydrostat, but it generally relies on the transfer of fluid in and out of cavities surrounded by muscular walls, as opposed to only tightly packed muscle^1^. Hydrostatic skeletons are responsible for motion in a wide variety of animals, including many worms, anemones, snails, and spiders^3^. One worm in particular, the velvet worm (Onychophora), uses a complex interplay of musculature and a hydrostatic skeletal system for locomotion, with the latter being the main antagonist for muscular actions. It extends, bends, and retracts its many unjointed legs (Fig. 1) using a set of muscles and the fixed volume of fluid within its body^10^. Within the field of soft robotics, hydrostatic skeletons and the motions they generate can be closely compared to many fluidic elastomer actuators due to their reliance on fluid movement for actuation, and they have motivated projects such as a rotary actuator inspired by spider legs^11^, a variable compliance gripper^12^, and this work.

**Fig. 2:**
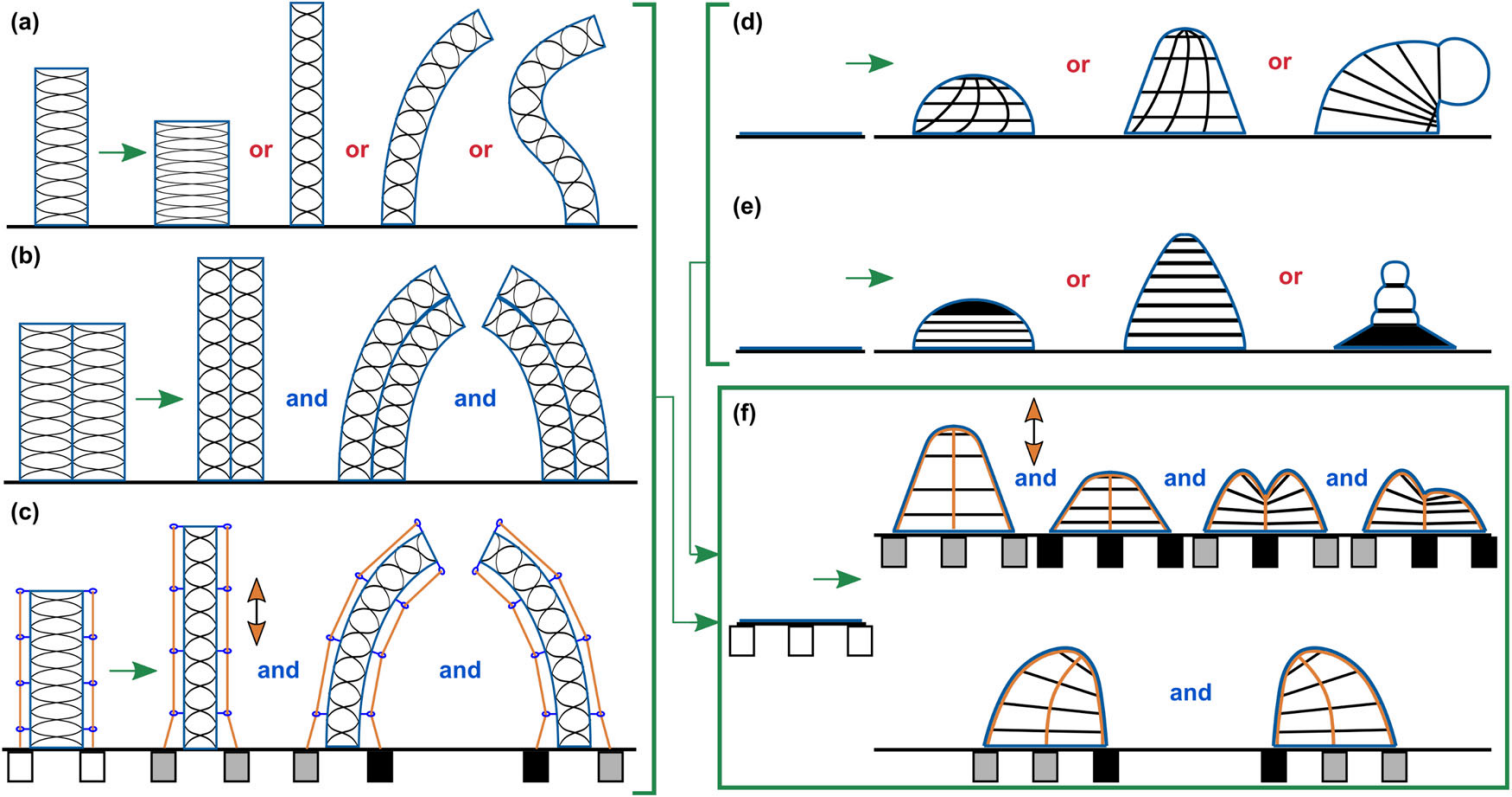
Comparison with existing fiber-reinforced inflatable actuators. **a** The majority of the literature presents cylindrical actuators that move along a one-DOF path defined during fabrication. **b** Placing these actuators in parallel can increase the deformation space by one DOF per actuator. **c** When tendons (orange) are added to these actuators to apply tension, the same active control can be accomplished with fewer pneumatic channels while allowing the actuator to both push and pull (orange arrows). Actuators that start as a flat membrane can also be found in the literature, either relying on **d** nonlinear fiber arrays or **e** fabrics to define their deformation space. Like most other fiber-reinforced actuators, their single DOF is defined during fabrication. **f** This work presents multiple-DOF, fluidic membrane actuators with a single fluid channel and embedded tendons that can produce many of the versatile deformations of a hydrostatic skeleton while more-fully utilizing the extensibility of elastomer. The servomotors shown in **c** and **f** are filled white to represent no applied tension, gray for low tension, and black for high tension.

Producing the motion of a velvet worm leg on a mobile soft robot using existing soft actuators is not a trivial task, and soft roboticists are actively working to better replicate biological systems^13^. Many attempts have been made to incorporate select capabilities of hydrostatic skeletons into soft robotic actuators. Fluid-driven soft actuators are commonly used to create extension, contraction, bending, and twisting deformations, often by relying on inextensible fibers to define their deformation space (e.g., McKibben actuators^14^, fiber-reinforced elastomeric enclosures^15^, and shape-matching, fiber-reinforced bending actuators^16^). While fibers can be arranged in a variety of configurations to create actuators with any combination of those deformations, such as in our previous work with soft actuators that utilize two fiber sets to create local planar strain coupling^17–19^, the motion of the resulting actuators is also generally constrained to a single active degree of freedom (DOF) that is defined when the actuator is fabricated and controlled by adjusting the pressure exerted on the elastomer. Fluidic elastomer actuators also often take a cylindrical or conical shape, extending or contracting axially and/or bending by curving that axis^20^ (Fig. 2a). When active control of these fluidic actuators is desired in additional DOFs (e.g., to make continuum robots^21,22^), other controllable elements must be added to the actuators (generally one per DOF). Some researchers have accomplished this by combining multiple fluidic elastomer actuators to control stiffness and create extension, bending, and twisting motions (e.g., refs. ^23–25^) (Fig. 2b). Others have done this by adding low-melting-point alloy channels^26^, active coiled nylon^27^, or motor-driven tendons^28–30^ (Fig. 2c), to name a few.

Soft robots actuated by motor-driven tendons, in particular, have proven to be highly versatile. These tendons can be routed internally or externally to the body^31^, often guided by inelastic, sometimes rigid components such as eyelets^8^ or spacer disks^32^. They have been used to drive a variety of soft robots including elastic foam robots^33,34^ and elastomer robots^35,36^. Some solutions, such as the STIFF-FLOP actuator with tendon-based stiffening^30^ and the OctArm manipulator^8^ allow for elastomer extension via inflation despite the presence of tendons, but the majority of tendon-driven elastomer actuators do not attempt high elastomer extension.

**Fig. 3:**
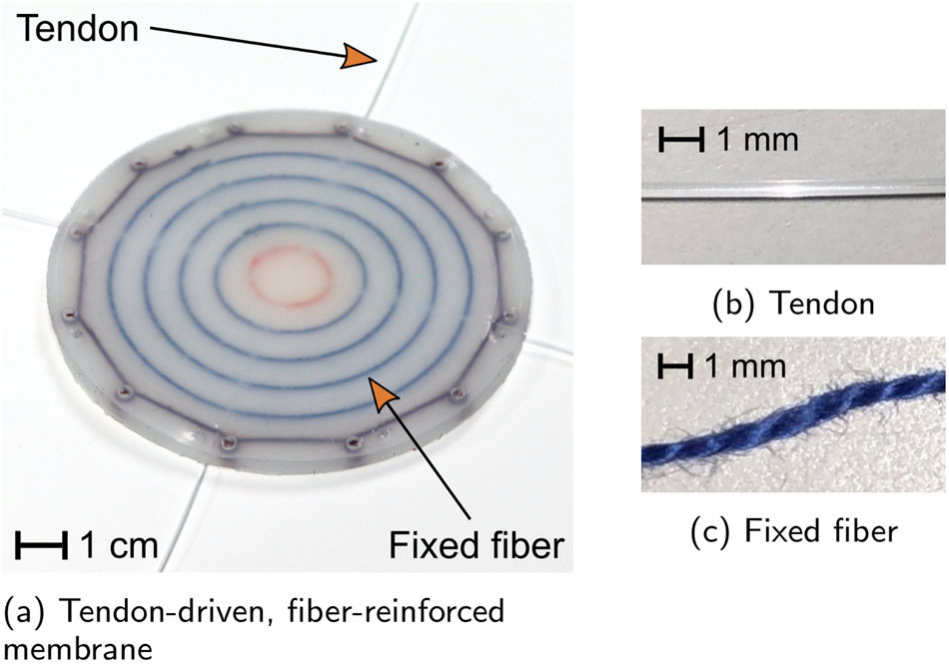
Sliding tendon and fixed fiber placement. **a** Tendon-driven, fiber-reinforced elastomer membrane. **b** The tendons are smooth and nonporous, allowing them to avoid bonding with the elastomer during fabrication. **c** Fixed fibers are frayed and can be penetrated by the elastomer, encouraging bonding.

In fact, while many soft actuators are built with elastomer, most of them do not take full advantage of the extensibility of modern elastomers. A commonly used elastomer in soft robotics, Ecoflex™ 00-30 (Smooth-On, Inc.), for example, is capable of stretching to 1000% its initial length (900% elongation at break^37^); yet, we rarely see elastomer actuators with multiple active DOFs that stretch the elastomer over 200%. Continuum robots that do extend significantly generally utilize a mechanism other than stretching elastomer by inflation, such as adding additional body material (e.g., refs. ^32,38,39^). Most other actuators that do utilize higher elastomer stretches do not attempt active control of more than one DOF (e.g., fiber-reinforced elastomer membranes^19^ (Fig. 2d) and 3D texture morphing inflatable membranes^40,41^ (Fig. 2e)).

In this article, we present a soft actuation technique that can produce many of the three-dimensional deformations seen in natural hydrostatic skeletons while more fully utilizing the extensibility of elastomer (Fig. 2f). Embedding tendons that can slide through an inflatable elastomer membrane lets the elastomer stretch in the direction of the tendon. This allows for high elastomer deformation compared to most other soft actuators while retaining the ability to actively extend, contract, and bend in multiple directions, much like a natural hydrostatic skeleton. A single membrane can also deform to grab objects from an initially planar configuration. If two or more of these membranes are used antagonistically, it is possible to use the proposed actuators with a fixed mass of fluid (e.g., precharged air^28^), negating the need for large, inefficient pumps or compressors during operation. We demonstrate this using fully soft, initially planar, fiber-reinforced elastomer membranes with radially aligned tendons. They are presented in detail, characterized, and installed on a simple wheeled platform where they show the ability to walk using a fixed mass of air with a gait similar to that of a velvet worm. The membranes can turn the vehicle, navigate uneven terrain, and climb up a ramp. When they are done with a task, they can then be deflated flush with the body of the soft robot to, for example, fit through small spaces or preserve the hydrodynamic profile of an underwater vehicle. The proposed technique can be applied to soft actuators used in a variety of robotics applications as, for example, soft appendages, control surfaces, and end effectors.

## Results

Here, we present and characterize a tendon-driven, fiber-reinforced elastomer membrane actuator that can exhibit high stretch while grasping small objects and controlling its shape like a natural hydrostatic skeleton. We then apply a pair of them on a simple wheeled platform to demonstrate walking locomotion similar to a velvet worm.

### Tendon-driven, fiber-reinforced elastomer membrane

One of the membranes fabricated to demonstrate the utility of the actuation method proposed in this article is shown in Fig. 3. Its two main features are fixed fibers arranged as a set of concentric rings and tendons anchored to the central fixed fiber that extend radially out of the edge of the elastomer. All of the fibers are embedded at the mid-plane of the elastomer membrane. The material of the fixed fibers, a cotton embroidery thread, was chosen both because it is frayed (increasing the contact area between the elastomer matrix and the fiber) and because the elastomer is able to penetrate and fill any gaps between the individual cotton fibers that compose the thread (making it necessary to tear the elastomer for the fiber to slide). Tendons, on the other hand, are chosen to reduce friction and bonding with the elastomer matrix. Here, a monofilament fishing line is used as the tendon, and mineral oil is added as a lubricant after the membrane is fabricated to further reduce friction. The membrane is 5 mm thick and 114 mm in diameter. It is clamped to a rigid plate by a clamp ring with an inner diameter of 94 mm and inflated with air in the following experiments.

### Membrane characterization

**Fig. 4:**
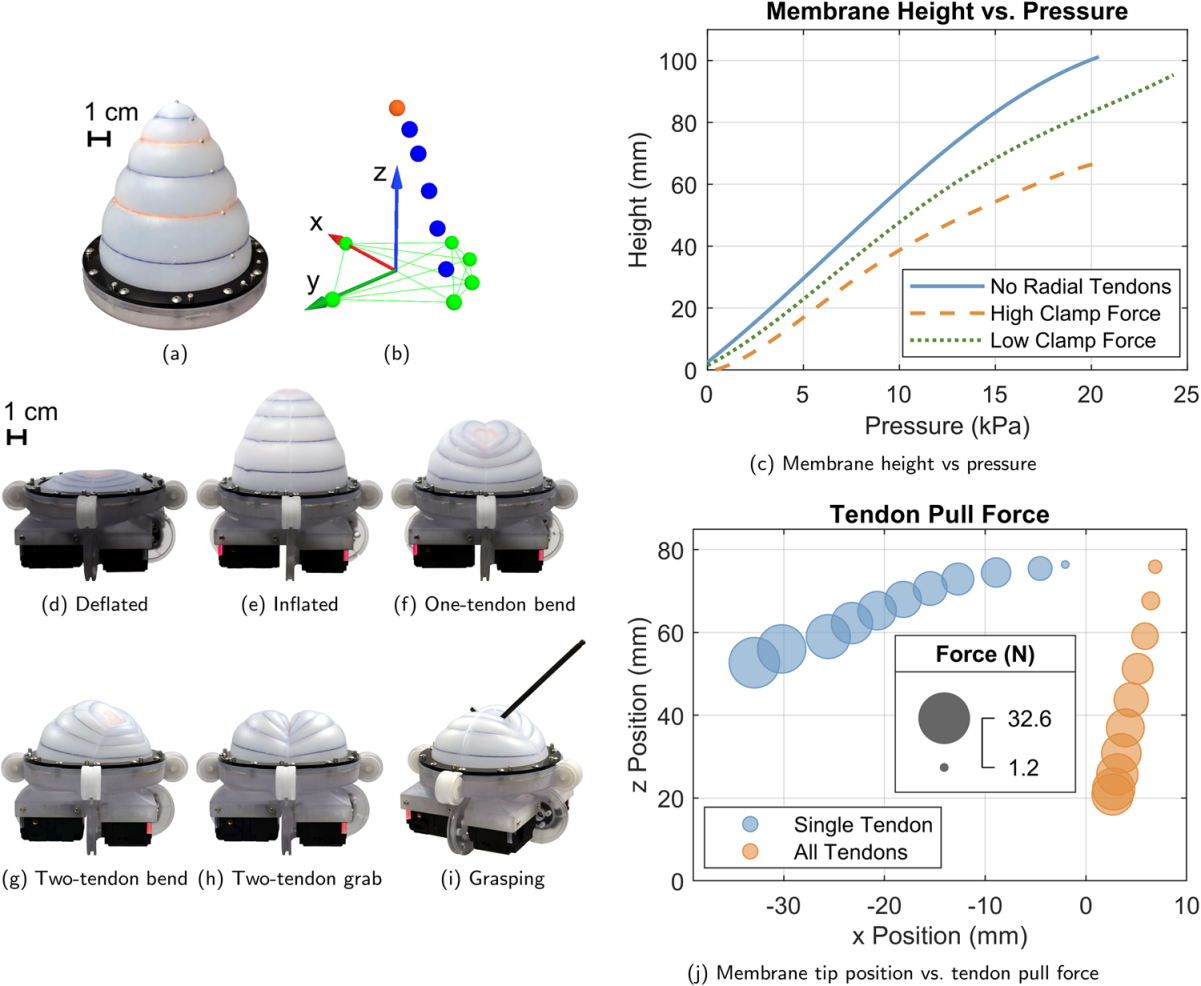
Membrane actuation characterization. **a** An inflated membrane without radial tendons is shown outfitted with motion capture markers next to **b** the measured coordinate system and markers. This instrumentation is used for **c** the inflation tests of two membranes, one with and one without radial tendons. Membrane height is measured using the motion capture marker at the center of the deflated membrane (orange, top), and extension along the membrane is measured by fitting a spline along the motion capture markers from the clamp to the highest marker. Intermediate motion capture markers (blue) are positioned directly over the intersection of tendons and fixed fibers, when present. **d**–**h** The membrane is then actuated by compressed air and servos to make various shapes. **i** Grasping is accomplished by holding an object in the two-tendon grab shape (demonstrated in Supplementary Movie 1). **j** The forces required to make variations of shapes **e** and **f** are also plotted.

Figure 4a–c shows extension testing of the membranes and Table 1 provides maximum values from those tests. Allowing tendons to slide through an elastomer body prevents the tendon from reducing the elastomer’s extensibility tangent to the fiber, only constraining the deformation of the membrane when tension is applied. The tendon-driven, fiber-reinforced membrane fabricated for this study is able to extend out of the initial plane of the membrane to 101% of its diameter at 23.9 kPa. A maximum measured elastomer stretch of 297% was recorded at 20.7 kPa along the path of one of the tendons.

**Table 1:** Tendon-driven, fiber-reinforced membrane characterization maximum values.

Radial Tendons	Clamp Force	Pressure	Out-Of-Plane Extension		Spline Stretch		
			Height	% Diameter	Δ*L*	% Stretch	MPSS
None	High	20.3 kPa	101.5 mm	108%	61.1 mm	240%	309%
4	Low	23.9 kPa	95.3 mm	101%			
4	High	20.7 kPa	68.1 mm	72%	42.6 mm	203%	297%

High clamping force is just sufficient to stop air from leaking under the clamp, while low clamping force allows for leaks. Spline stretch describes the stretch of the elastomer along the path of a radial fiber estimated by a spline between motion capture markers. MPSS is the maximum percentage stretch for the spline between two consecutive motion capture markers.

Friction between the tendon and the elastomer matrix limits the extension of the elastomer. This is especially true when the clamp is applying pressure because the tendons slide under the clamp. As shown in Fig. 4c, reducing the clamping force increases the extension for a given pressure, but it also allows for air to leak out from under the membrane, hindering the membrane’s ability to be used in an antagonistic pair with a fixed mass of air. Lubricating the tendons reduces friction, and it is also possible to embed stiff sheaths around the tendon at the clamp location to reduce the pressure on the tendon. The friction at high clamping force reduces the membrane’s out-of-plane extension by 36% and its maximum measured elastomer stretch by 12% compared to the membrane with no radial fibers.

To automate the actuation of the tendons, a fixture was designed that incorporates a servo at the end of each of the four tendons to control their length or tension. The membrane can then be set to different geometries to accomplish tasks such as pushing, pulling, bending, and grasping by coordinating pulls on the tendons (Fig. 4d–i). Pushing is accomplished by releasing the tension on the tendons and increasing the inflation pressure. Pulling can be achieved by actuating the tendons with equal tensions, either with or without venting the air inside the membrane. Actuating the tendons with different tensions allows for bending motions, e.g., in the direction of a single tendon (Fig. 4f) or between two of them (Fig. 4g). Pulling two opposite tendons while releasing the other two creates a crease in the membrane that can be used to grab small objects (Fig. 4h), such as a 13 g hex key (Fig. 4i and Supplementary Movie 1).

The tendon pull forces required to create some of the deformations in Fig. 4e and f are shown in Fig. 4j. Membrane tip location in the X-Z plane is plotted versus the tendon pull force for a single-tendon bend (Fig. 4f) and an equal pull of all four tendons (Fig. 4e). After inflating the membrane to 16.9 kPa, a force of 32.6 N is required to retract a single tendon to its initial embedded length while the other tendons are unconstrained. Pulling all of the tendons equally to their initial embedded lengths requires 22.0 N per tendon at 16.4 kPa.

### Membrane locomotion

**Fig. 5:**
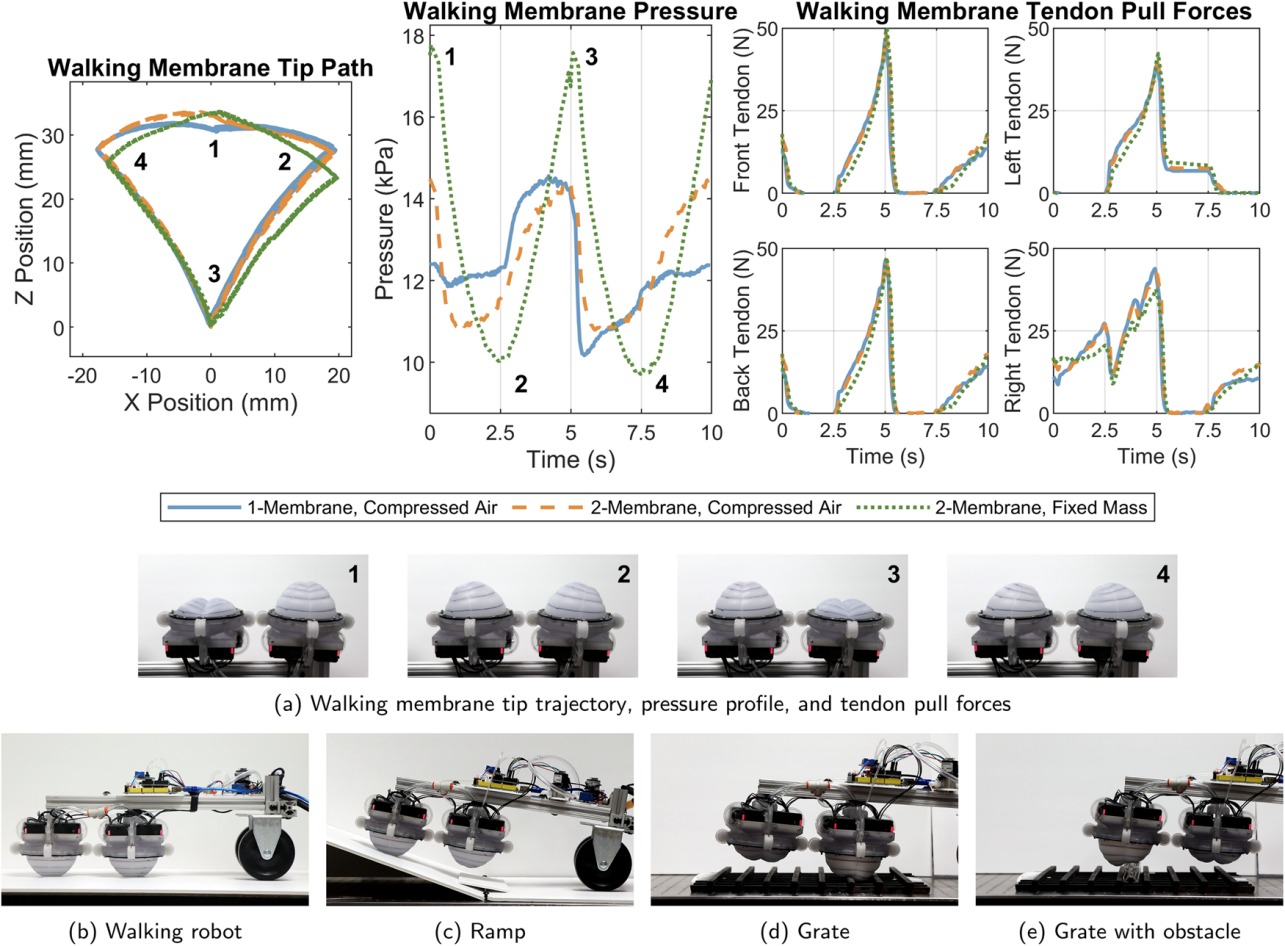
Membrane locomotion testing. **a** Characterization of the motion used during walking membrane locomotion. The tip path shows the raw data from five step motions for each pneumatic configuration, while the pressure and pull force plots show the time-synchronous average over all five steps. The four programmed positions (of the numbered membrane in the images) are numbered 1–4 in each plot and image. **b** The membranes are used to walk on a flat surface, **c** up a 20^∘^ ramp, **d** over a grid of 10 mm square metal bars (total height 20 mm), and **e** over an obstacle (25.4 mm aluminum extrusion (80/20^®^)) on the metal grid.

If the membrane is oriented to face a surface, the membrane’s tip can be moved up to the surface and used to exert a force back on the clamp, moving the assembly across the surface in a walking-like fashion (Supplementary Movie 2). Multiple membranes following the same tip trajectory at different phases can act as sets of legs, walking with a gait similar to that of a velvet worm^10^. To demonstrate this, a tip trajectory designated by four sets of tendon lengths (as read by the servo, neglecting tendon extensibility) was designed to function as a single step in the velvet worm’s walking locomotion. The trajectory of the tip without loading and the average pressure profile per step are shown in Fig. 5a for three different pneumatic configurations:

1. a single membrane connected to a constant compressed air source;
2. two membranes pneumatically connected in parallel to a constant compressed air source, with the second membrane following the same path 180^∘^ out of phase; and
3. two membranes pneumatically connected in parallel, inflated, then disconnected from the compressed air source to fix the mass of air in the system, also with the second membrane 180^∘^ out of phase.

Each pneumatic configuration produces a unique, repeatable tip path and pressure profile. The single-membrane configuration with a constant air supply produces a nearly symmetrical tip path with a distinct dip at the first set of tendon lengths (denoted by 1 in Fig. 5a). When a second membrane is connected in parallel to the same air source and actuated 180^∘^ out of phase, it collapses to position 3 while the first membrane is moving to position 1. This creates a higher pressure at position 1 for each membrane than in the first pneumatic configuration, pushing the tip higher at position 1 and causing an asymmetry between the path from positions 4 to 1 and 1 to 2. The path from positions 1 to 4, however, remains largely unaffected.

Closing a valve to fix the mass of air within the dual-membrane system has a noticeable impact on the tip path and a larger impact on the pressure profile. Because air can no longer enter and leave the system (despite moving freely between the membranes), large pressure spikes are generated at positions 1 and 3 (where the desired system volume is lowest and the air is more highly compressed by the membrane) and the pressure dips lower than the other configurations at positions 2 and 4 (where the desired system volume is highest and the air is the least compressed by the membrane). This pressure profile change lowers the tip at positions 2 and 4 while pushing the tip up at position 1. While the tip trajectory used in these experiments was chosen to maximize walking performance across the various pneumatic configurations presented, it would be possible to mitigate these pressure spikes by adjusting the programmed tip trajectory to ensure a constant volume between the two membranes. This would be especially important if an incompressible fluid were chosen as the driving fluid instead of air.

To evaluate these membranes’ utility as legs in a walking robot, two of them are mounted on a simple wheeled platform (Fig. 5b) and used to move across a flat surface, up a ramp (Fig. 5c), and over uneven terrain (Fig. 5d, e). The robot has a total mass of 3.5 kg, putting a load of up to 1.4 kg on the front membrane and up to 2.3 kg on the rear membrane on level ground, due to their varying distance from the wheels. Each step has a length of 37 mm when the membrane is not in contact with a surface, but the useful forward motion per step is reduced in practice depending on the membrane load and terrain. In practice, with a constant supply of air on flat ground, the walking robot maintains an average forward walking speed of 12.4 mm s^−1^ (13.2% of the membrane’s diameter) or a turning speed of 2^∘^ s^−1^ when each membrane’s step is set to take 3 s with a maximum tendon pull rate of 91.9 mm s^−1^. The robot can move up a 20^∘^ incline starting from a flat surface, provided the membranes are able to reach the ground during the transition between the flat and inclined surfaces. It is also able to walk across a grid of stacked 10 mm square metal bars (Fig. 5d, e) and over a piece of 25.4 mm aluminum extrusion (80/20^®^) placed on the center of that grid (Fig. 5e). When the robot is set to use a fixed mass of air with the same programmed gait as the other walking experiments, its walking speed decreases to 5.4 mm s^−1^ and it is unable to complete the other maneuvers.

## Discussion

The grasping capabilities of this membrane are shown here as a proof of concept. If the membranes are designed specifically for grasping, the tendon and fixed fiber patterns can be tuned to maximize the deformation in the grab configuration (Fig. 4h). The overall shape of the membrane could also be changed (e.g., a three-dimensional starting configuration as opposed to the circular disc presented). In the presented configuration, the tendons pull down on the fixed fibers, limiting the membrane’s upward deformation and its ability to wrap around an object. Changing the fixed fiber pattern from concentric rings to a pattern that does not connect along the line of two opposite tendons could allow for better grasping performance. Additional tendons (possibly with termination points other than the central fixed fiber) could be added to more precisely control the grasping force or the distance between the closed sides of the membrane.

Manipulation tasks could also be accomplished by embedding an attachment point (much like the embedded, threaded insert used to mount our cephalopod-inspired suction cups to rigid measurement equipment in ref. ^42^) within the membrane’s innermost fixed fiber. Threading the tendons through that attachment point would allow the tendons to directly manipulate the payload. This could enable precise positioning of the payload, potentially to point an attached camera or laser, flip switches, or press buttons.

A variety of factors dictate the maximum actuation speed of the membranes tested in this article. The maximum flow rate of the compressed air supply places an upper limit on the inflation speed of the membranes. Friction between the tendons and the elastomer is low enough that the limiting factor on tendon pull speed is either the inflation rate or the maximum speed of the servos. Viscoelasticity also affects the rate at which the elastomer may be deformed, but fluidic elastomer actuators have been shown to actuate at 4 Hz with large-amplitude motion^43^, suggesting that the air supply or servos would likely be the limiting factor for these membranes.

When these membranes are applied as legs on a vehicle, it is possible to change both the position and the attitude of the vehicle by changing the shape of the membranes. This is true for all six DOFs when at least three membranes are used. Adjusting the stiffness of the membranes for a given position and attitude would also provide a tunable vehicle suspension, allowing for programmable shock absorption and rejection of various vibrations from the terrain. A vehicle with six membrane legs would be controllable in six DOFs without additional support or complicated stability considerations. It would also be able to use its two sets of three legs as antagonistic pairs for operation without a compressed air supply, as long as a sufficient quantity of air can be supplied to inflate all of the membranes at once before isolating the pneumatic circuit.

The vehicle used in the locomotion demonstration could be improved in a variety of ways to increase its versatility. Currently, the tendon lengths are fed to each membrane without consideration for the terrain or obstacle encountered. Utilizing the servo load feedback as a membrane load sensor could improve walking performance in the event of a sharp slope change by alerting the controller that additional extension is required to reach the ground for a successful step. When there is no load on the membrane, the tendons experience a tension that is a function of the inflation pressure. Adding a compressive load to the membrane (like the force against the ground) reduces the tension in the tendons, which can be read by the servos. It would also be possible to use optical fibers as the tendons^44^ or mount a camera on the clamp under the membrane to track its geometry and estimate loads^45^ for the same purpose. Alternatively, if a set of these membranes were mounted on a continuum robot body in a similar layout as a velvet worm’s legs, the robot would be able to navigate complex terrain by repositioning the membranes, as necessary.

Such feedback could also improve the walking robot’s performance in the fixed-mass case. Its lower speed and inability to complete the maneuvers in Fig. 5c–e when the compressed air supply is deactivated is likely due to the robot’s lack of compensation for the drops in system pressure at positions 2 and 4 in Fig. 5a. The manifestation of these pressure drops is a notable dip forward in the robot’s gait that prevents it from clearing raised obstacles or changes in slope. These pressure dips could either be mitigated by increasing the mass of air shared between the two bladders (by increasing the system’s starting pressure or adding an additional bladder as an accumulator) or by allowing for active control of the desired tendon lengths to compensate for reductions in pressure and walking performance.

The fixed-mass case presented for the locomotion demonstration is uncommon for fluidic elastomer actuators because it uses a constant quantity of air to accomplish a task. Most extending fluidic elastomer actuators rely on venting fluid to reverse their actuation, sometimes while an antagonistic muscle is inflated by an external source. Any energy expended to pressurize the working fluid is then lost in this venting procedure, making those fluidic elastomer actuators impractical for many small mobile platforms. The actuators presented in this article, however, are able to actively move fluid from one actuator to another by pulling or releasing the tendons, as is done in ref. ^28^. In the locomotion demonstration, for example, as one membrane in a pair is contracted, its fluid is pushed into the second membrane to inflate it, much like the action of a hydrostatic skeleton. If it is desirable to vent the fixed mass of air to deflate the actuators, there are a variety of options for replenishing the fluid required for operation (e.g., liquid-gas phase change^46^, electro-pneumatic pumps^47^, microcompressors, compressed air cylinders, and chemical reactions^48^). It would even be possible to configure these actuators to act as their own pumps, similar to the technique proposed by ref. ^49^.

Another advantage of the form factor of these actuators is their ability to deflate from a potentially complex 3D geometry down to a plane. A common example of the utility of soft robots is their ability to collapse in size or squeeze through small spaces, similar to how an octopus can squeeze through any hole larger than its beak, but many of the soft robots in the literature begin with an initially cylindrical shape that cannot collapse any further. The membranes in this article could be used as deployable structures wherever compact packaging is of the essence (e.g., spacecraft) and can fully retract against the body of a robot without using any additional internal space. Continuum surgical robots could deflate these membranes to fit through tight spaces before inflating them for navigation, manipulation tasks, or anchoring the robot in place. For example, the elastomer balloons used in ref. ^50^ to improve safety during colonoscopies could be augmented with this technique to enhance their ability to navigate through the colon, possibly even pulling the surgical instrument through the colon instead of pushing it through externally. Underwater robots such as our group’s CephaloBot autonomous underwater vehicle^51^, for example, could maintain an efficient hydrodynamic shape (to reduce drag) until a manipulator is necessary, at which point multiple appendages could inflate from the body of the robot to manipulate an object. This is especially useful for a vehicle like the CephaloBot that has a distributed sensory system^52,53^ that could be adversely affected by the fluid effects that would manifest as a result of protrusions from the vehicle (e.g., vortex shedding). The membranes could also be inflated with fluids of various densities to help the CephaloBot’s fiber-reinforced membrane variable buoyancy system^54^ maintain neutral buoyancy while manipulating objects.

Finally, these actuators demonstrate the ability to utilize a large portion of the elastomer’s extensibility while retaining control of multiple DOFs. The results in Table 1 show that these membranes can stretch up to 297% directly over a tendon before risking the fixed fibers separating from the elastomer. This number could easily be increased by changing the orientation of the fixed fibers so that they are not directly perpendicular to the direction of the largest stretch, depending on the application. Fixed fibers could also be omitted entirely if the loads on the tendons are sufficiently low to prevent tearing through the elastomer. If there are no fixed fibers that need to maintain a bond with the elastomer, the membrane could be actuated up to the limits of the elastomer, itself.

## Conclusions

In this article, we demonstrate a technique for embedding sliding tendons in inflatable, fiber-reinforced elastomer membranes to enable soft roboticists to better utilize the extensibility of elastomer and to mimic many of the motions of a natural hydrostatic skeleton in their soft actuators. The example membrane actuator presented here can inflate from a planar configuration to a variety of three-dimensional shapes by actively extending, contracting, and bending in multiple directions, all with only one fluid cavity. During this deformation, it can stretch to nearly three times its initial length directly along the path of a sliding tendon. Because the membranes can be used to both push (via inflation) and pull (by pulling the tendons), pairs of them can accomplish many of these motions using a fixed mass of air, negating the need for a compressed air source. A single membrane can be used to grasp objects, and multiple membranes can be used together to walk with a gait similar to that of a velvet worm on flat surfaces, climb a ramp, and navigate uneven terrain.

Future work on this actuation technique will involve investigating other choices for the fixed fiber material to avoid some of the fiber bonding issues at high elastomer strains presented in the Supplementary Note. It will also explore methods for creating and anchoring more complex tendon patterns within the elastomer (e.g., curved tendon patterns to allow for torsional deformation, as in ref. ^19^) and other materials or actuation methods to improve or replace the tendons (e.g., shape memory alloy wires^55,56^), servos, and clamping mechanism (e.g., fully embedding the membrane in an elastomer body).

## Methods

Here, we outline the membrane fabrication procedure, the membrane clamping and control hardware, and the experimental procedure for the membrane characterization tests.

### Membrane fabrication

**Fig. 6:**
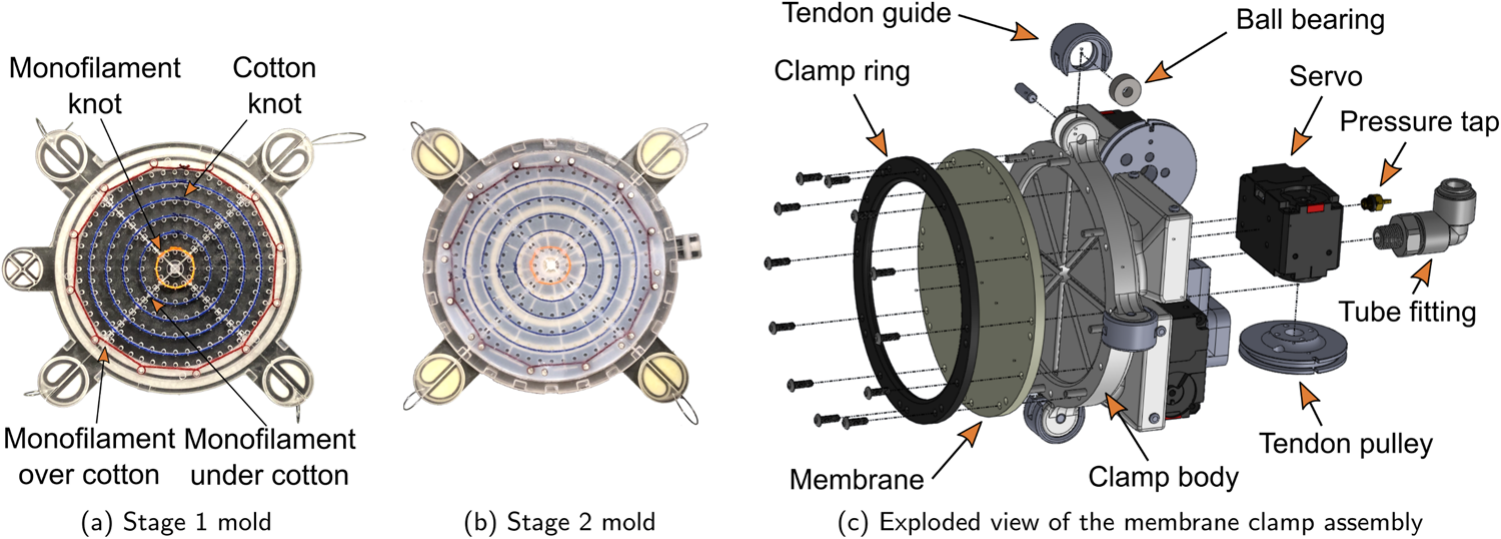
Tendon-driven, fiber-reinforced membrane molds and clamping fixture. **a** Fibers and tendons are arranged on a 3D printed template that also serves as the first of two molds. Cotton fibers are used where bonding with the elastomer matrix is desired, and a monofilament is used for the tendons where bonding is to be avoided. **b** The product of the first mold is inserted into a second mold where the holes from the guide posts are filled in. **c** The finished membrane is clamped to an assembly with servos that actuate each tendon.

The membranes in this study were fabricated using a two-stage molding process similar to that presented in our previous work^19^. Six cotton fibers (DMC 6-strand cotton embroidery floss) and two monofilament tendons (Sufix Advance monofilament fishing line, 25 lb test) are placed on a 3D-printed (VisiJet Armor, 3D Systems ProJet MJP 2500 Plus) template in the desired pattern (Fig. 6a), resulting in five concentric rings of fixed cotton fibers, four individually controllable tendons composed of two knotted lengths of monofilament, and an additional circular cotton fiber that sits under the clamp to prevent the elastomer from pulling out of the clamp. Square knots were used to join the ends of the cotton fibers to form rings, and simple overhand knots were used to anchor the monofilament tendons to the innermost cotton ring. Any required cotton knots were reinforced with cyanoacrylate glue (Loctite Ultra Liquid Control) to prevent the knots from untying inside the finished sample. Any excess monofilament that extended out of the finished membrane was wrapped around posts during fabrication to maintain tension throughout the molding process. Mixed and degassed elastomer (Ecoflex™ 00-30, Smooth-On, Inc.) was poured over the fibers and tendons, and the template was placed in a vacuum chamber to ensure a stronger mechanical bond with the cotton fibers by pulling elastomer between the strands of cotton. After the first stage, the sample was placed in a second-stage mold (Fig. 6b) where the holes from the first-stage guide posts were filled with elastomer. Any weak adhesion between the elastomer and the monofilament tendons can be easily broken when the membrane is first inflated.

To prevent the tendons from tearing through the elastomer at high loads, they are routed either above or below the fixed fibers to transmit the load to the fixed fibers instead of the elastomer matrix. In Fig. 6a, the tendon is routed above the outermost fixed fiber (the clamp fiber) and below the other fixed fibers. Once the membrane is fully fabricated, the face shown is placed on to the clamping fixture, and a clamp ring is fastened to the opposite side (the clamp is shown in detail in Fig. 6c). When inflated, this places the tendon on the low-pressure side of the membrane. Pulling on the tendons exerts a force on the membrane and fixed fibers opposite the force of the internal pressure, allowing the tension in the fixed fibers to support some of that pull force. Otherwise, the tendon would likely pull through the elastomer matrix, causing the membrane to fail. The same effect happens at the clamp fiber. As the tendon pulls away from the clamp, the clamp fiber helps to support the tendon instead of allowing it to tear through the elastomer and rub against the clamp ring. In cases where the tendon exerts forces tangential to the fixed fibers, as in twisting motions, the fixed fiber can be looped around the tendon, creating a guide for the tendon. No tendon-caused membrane failures were observed during testing using this tendon-routing method except for when there was friction against an obstacle, as described in the Supplementary Note.

### Membrane control hardware

An exploded view of the clamp assembly used to secure and actuate the membranes tested in this chapter is shown in Fig. 6c. The clamp body, tendon guides, and tendon pulleys were 3D printed (VisiJet Armor, 3D Systems ProJet MJP 2500 Plus), and the clamp ring was laser cut (ULS PLS6MW, 50 W CO_2_ Laser) from delrin and filed to smooth the inside edge. Four Dynamixel XL430-W250-T servos, one for each tendon, are fastened to the clamp body. Each servo provides current and position feedback and is capable of exerting a maximum force of 75 N or a maximum pull rate of 128 mm s^−1^ on a tendon via a 20 mm radius pulley. The servos are controlled by an Arduino Mega through a Dynamixel Shield, and membrane inflation pressure feedback is provided by an absolute pressure sensor (MPXHZ6250AC6T1, Freescale Semiconductor) sampled by a 16-bit ADC (MAX1167BEEE+, Maxim Integrated Products). Automated inflation and deflation valves are connected to the pneumatic circuit to prevent accidental damage to the membrane and to ensure that the tendons are always held taut to prevent tendon bunching (see Supplementary Note).

### Membrane characterization tests

Membrane extension testing was conducted on membranes with and without radial tendons. All of the membranes had the same dimensions and configuration of fixed fibers. Tests were conducted with the tendons detached from the servos, leaving them free to slide through the membrane. The membrane was clamped down and inflated to the maximum tested pressure repeatedly to allow the membrane to settle in the clamp. Some elastomer pulls out from under the clamp when first inflated, causing the membrane to bow slightly upwards when deflated, as seen in Fig. 4d. The maximum pressure was chosen to prevent the fixed fibers from separating from the elastomer matrix during the test.

Motion capture markers were placed on the membrane along the radius (along a tendon, if applicable) where it intersected with the fixed fibers, as shown in Fig. 4c. The clamp ring (Fig. 6c) was also outfitted with motion capture markers to provide the coordinate system for measurement. A Qualisys motion capture system with 5 cameras was used to capture the 3D position of each marker as the membrane was slowly inflated from 0 kPa to the maximum tested pressure.

Height, as reported, is defined as the displacement of the motion capture marker placed at the center of the deflated membrane normal to the plane of the clamp. The initially bowed state of the membrane, described above, is recorded as a height of zero for this measurement. The height curves reported in Fig. 4c are Fourier series fit to the motion capture data with R^2^ values above 0.998, in all cases. Table 1 shows the maximum obtained heights, as well as the ratio of the maximum height to the membrane’s diameter in the clamp under out-of-plane extension.

Spline stretch is also reported from the same inflation data used to determine out-of-plane extension. These values describe the stretch of the elastomer along the membrane. To determine this, a spline is fit to the motion capture markers on the membrane using MATLAB’s spline function. The length of this spline is integrated at each time step and the difference between it and the spline’s length at 0 kPa is reported as Δ*L*. Percent stretch is the ratio of the maximum spline length to its initial length. The maximum percentage stretch of the spline (MPSS) between two consecutive motion capture markers is determined in the same fashion.

The pull forces reported in Fig. 4j were estimated from the current draw read from the servos and their rated stall torque. To collect this data, the membrane was prepared as described above and inflated with all the tendons set to equal embedded lengths. In one case, a single tendon was then pulled incrementally while the other tendon servos were deactivated. In another, all four tendons were pulled simultaneously in the same increments. A delay was added at each step to allow for the membrane’s pressure to reach a steady state. The tip position and servo current (for all active servos) were averaged at each step, and the servo current was converted to pull force assuming a linear torque response to the applied current up to the stall torque.

### Supplementary information

Supplementary Information

Description of Additional Supplementary Files

Supplementary Movie 1

Supplementary Movie 2

## Data Availability

The data supporting the findings of this study are available from the corresponding author upon reasonable request.
