# Supplementary material for: High-stretch, tendon-driven, fiber-reinforced membrane soft actuators with multiple active degrees of freedom
Source: Commun Eng. 2024 Feb 23;3:25. doi: 10.1038/s44172-023-00139-3 (PMC10955944; doi:10.1038/s44172-023-00139-3)
Supplement: Supplementary file 1 — Supplementary Information [file 44172_2023_139_MOESM1_ESM.pdf]

Supplementary Note: Tendon-Driven, Fiber-Reinforced Elastomer Membrane Failure Modes

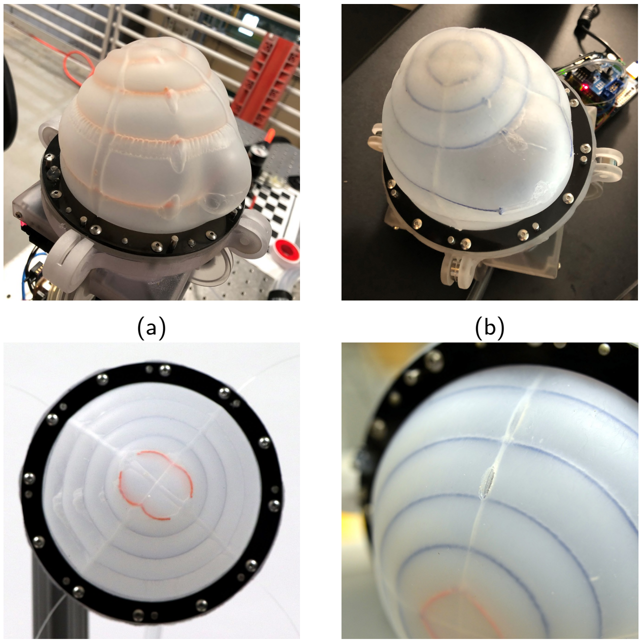

(a) (b) (c) (d)

Fig. S1: **Examples of tendon-driven, fiber-reinforced membrane failures.** Fixed fibers can cause failure when **a**, they tear through the elastomer, **b**, their knots untie in the membrane, or **c**, they break at an intersection with a tendon. All fixed-fiber failures shown here happened when the membrane was over inflated ($\geq$ 20 kPa). The only elastomer damage from a tendon resulting in a leak occurred in **d**, where elastomer was trapped between a tendon and an obstacle, causing a hole to tear in the membrane at that point.

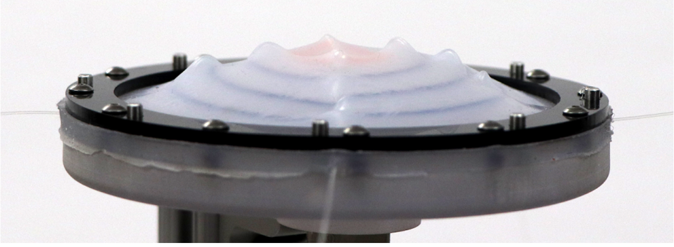

Fig. S2: **Tendon behavior without applied tension after deflation.** If the tendons are pulled into the membrane during inflation and tension is not applied during deflation, the tendons bunch up within the membrane.

While the actuation technique presented in this article has great potential in soft robotics, there are several engineering issues that can be addressed to improve the durability of the membrane actuators proposed. Most failures of these membranes occurred when the fixed fibers tore through the elastomer, as shown in Fig. S1. The most common type of failure is when a fixed fiber fully tears through the elastomer membrane, allowing the membrane to balloon outwards at that location. While this usually starts at the location of a knot, where elastomer is unable to penetrate the dried adhesive used to prevent the knots from untying, it can occur anywhere along the length of the fixed fiber, like the failure in Fig. S1a. When glue is not used over the knots, they sometimes untie at high pressures because the ends of the knots are cut very short to minimize the amount of embedded material (Fig. S1b). In one case (Fig. S1c), the tendon broke the center fixed fiber at the monofilament knot during a single-fiber pull at high pressure ($\geq$ 20 kPa). All of these failures could potentially be addressed by choosing a different material for the fixed fibers (e.g., a braided fishing line), by choosing a slightly stiffer elastomer, or by increasing the thickness of the membrane.

The tendons only broke through the membrane at high-friction locations, such as where a tendon repeatedly actuated against an obstacle or against the edge of the clamp. During a locomotion test with the metal grid, the front membrane repeatedly actuated against a sharp metal edge, helping the tendon cut through the membrane and causing a small leak (Fig. S1d). While this leak prevented a fixed mass of air from being used, the membrane still functioned with a compressed air source to compensate for the lost air. The clamp never caused a leak in the membrane, but the tendons did sometimes split the elastomer at the inside edge of the clamp. These issues at tendons could be addressed by embedding a semi-rigid sheath around the tendon under the clamp or by changing the elastomer or membrane thickness, as mentioned above.

Fortunately, none of the observed failures ever resulted in any catastrophic performance degradation or dangerous depressurization. Instead, the membranes failed gracefully, either operating normally with some form of deformity (Figs. S1a to S1c) or simply requiring some extra compressed air to compensate for a leak (Fig. S1d). Depending on the task at hand, a membrane with a failure may still function as desired. For example, the membrane in Fig. S1d was successfully and repeatedly used for locomotion demonstrations after failure. If the robot were operating with a fixed mass of compressed air and no additional compressed air source was available, however, that failure could have crippled the robot. Because of the relatively low air pressures ($<$ 25 kPa) used to actuate these membranes, none of them ever popped, and there was no observed damage to the clamp fixture.

Inflating these membranes pulls a length of tendon into the membrane to fill the expanding elastomer channel. This length of tendon does not freely slide out of the membrane during deflation. The tendon-driven membrane controller always attempts to maintain tension within the tendons, but if it fails or the servos are not attached, the membrane behaves as shown in Fig. S2. While the membrane remained functional and appeared undamaged after repetitive bunching of the tendons, it may have started to tear the elastomer channel around the tendon.
