# Supplementary material for: High-stretch, tendon-driven, fiber-reinforced membrane soft actuators with multiple active degrees of freedom
Source: Commun Eng. 2024 Feb 23;3:25. doi: 10.1038/s44172-023-00139-3 (PMC10955944; doi:10.1038/s44172-023-00139-3)
Supplement: Supplementary file 2 — Description of Additional Supplementary Files [file 44172_2023_139_MOESM2_ESM.pdf]

Description of Additional Supplementary Files

File name: Supplementary Movie 1

Description: Membrane grasping demonstration.

File name: Supplementary Movie 2

Description: Membrane locomotion demonstration.
